# Phytotoxic Effects and Microbial Responses to Ciprofloxacin and Its Removal by *Hydrilla verticillata*

**DOI:** 10.3390/toxics13100882

**Published:** 2025-10-16

**Authors:** Linzhi Lu, Rong Huang, Liang Wan, Guijia Li, Zhenhao Xu, Jiahao Guo

**Affiliations:** 1 Hubei Key Laboratory of Environmental Geotechnology and Ecological Remediation for Lake & River, School of Civil Engineering, Architecture and Environment, Hubei University of Technology, Wuhan 430068, China; 2 Key Laboratory of Intelligent Health Perception and Ecological Restoration of Rivers and Lakes, Ministry of Education, Hubei University of Technology, Wuhan 430068, China; 3 Innovation Demonstration Base of Ecological Environment Geotechnical and Ecological Restoration of Rivers and Lakes, Hubei University of Technology, Wuhan 430068, China

**Keywords:** ciprofloxacin (CIP), *Hydrilla verticillata*, oxidative stress, phytoremediation, microbial community

## Abstract

Ciprofloxacin (CIP), a widely used fluoroquinolone antibiotic, is frequently detected in aquatic environments, raising concerns over its ecological risks. In this study, the submerged macrophyte *Hydrilla verticillata* was employed to investigate its capacity for CIP removal and the associated ecotoxicological effects. A series of batch experiments were conducted to evaluate plant growth, photosynthetic efficiency, oxidative stress responses, CIP biodegradation pathways, and shifts in epiphytic microbial communities. Results showed that CIP significantly inhibited the growth of *H. verticillata*, with inhibition rates of plant length and fresh weight reaching 15.8% and 29.7%, respectively, at 5 mg/L. Photosynthetic parameters were severely suppressed. Fv/Fm represented the maximum quantum efficiency of PSII, significantly decreased by 94.3% at 10 mg/L, while chlorophyll a and b contents declined by up to 36.1% and 31.2%, respectively, compared to control. Antioxidant responses showed *H. verticillata* undergo peroxidation damage. Biodegradation analysis revealed that *H. verticillata* effectively removed CIP from water, with maximum removal rates of 37% at 1 mg/L and 31% at 5 mg/L under high biomass (4.2 g) conditions. CIP accumulation was higher in stems than in leaves, and three biodegradation intermediates (C306, C263, and C248) were identified, suggesting a degradation pathway involving piperazine ring cleavage, de-ethylation, and deamination. High-throughput sequencing further indicated that CIP exposure reduced bacterial diversity and richness on *H. verticillata* surfaces, while promoting antibiotic-resistant taxa such as *Actinobacteria* and *Bacteroidota*. These findings highlight the potential role of *H. verticillata* in antibiotic-contaminated water remediation.

## 1. Introduction

Global antibiotic production and consumption have risen sharply in recent years. Human antibiotic use nearly doubled from 2018 to 2023, escalating from 40.1 billion defined daily doses (DDD) to 75.1 billion DDD [1,2]. Similarly, agricultural and aquaculture antibiotics are projected to increase from 99,502 tons in 2020 to over 107,000 tons by 2030 [3]. However, conventional wastewater treatment plants remove only limited amounts of antibiotics, leading to their continuous release and pseudo-persistence in aquatic environments [4,5]. Consequently, chronic low-dose exposure poses substantial ecotoxicological risks to aquatic organisms and threatens ecosystem structure and function [6].

As primary producers in shallow lakes, submerged macrophytes play a central role in organic matter production through light-driven photosynthesis. They also provide essential surfaces and substrates for epiphytic biofilms composed of algae, bacteria, and other microorganisms [7]. Importantly, submerged macrophytes contribute to nutrient cycling, pollutant removal, water quality improvement, and overall ecosystem stability [8]. However, direct exposure to antibiotics may disrupt plant physiology, leading to inhibited growth, reduced photosynthetic activity, and altered metabolic profiles. Such phytotoxic effects not only impair the ecological balance but also may weaken the pollutant-removal capacity of aquatic macrophytes [9]. Although several studies have explored antibiotic toxicity in submerged macrophytes, research remains fragmented and species coverage is limited. For example, enrofloxacin inhibited the growth of *Vallisneria spiralis* while simultaneously enhancing phosphorus uptake from water [10]. Exposure of *Myriophyllum aquaticum* to tetracycline suppressed plant growth and elevated MDA and antioxidant enzyme activity [11]. Likewise, sulfonamides disrupted the growth and photosynthesis of *Vallisneria natans* [12], and oxytetracycline and sulfadiazine altered physiological responses in *V. natans* and associated biofilms [13]. These studies indicate that antibiotics can trigger overproduction of reactive oxygen species (ROS) and disrupt photosynthetic efficiency, leading to cellular damage. However, interspecific variation in tolerance has also been observed, suggesting differences in antioxidant defense capacity among species. Despite its global prevalence and ecological importance, the response of *H. verticillata* to ciprofloxacin has not been systematically investigated.

Submerged macrophytes such as *H. verticillata* and *V. natans* are widely applied in phytoremediation due to their ability to improve water quality in eutrophic lakes by removing nitrogen and phosphorus from both water columns and sediments [14]. Phytoremediation is cost-effective, environmentally sustainable, and scalable. Nevertheless, its efficacy may be limited by plant sensitivity to contaminants such as antibiotics, which can reduce growth and pollutant uptake efficiency [15]. The ability of macrophytes to accumulate and metabolize antibiotics is a key determinant of their tolerance and remediation potential. Despite their proven ability to reduce antibiotic concentrations in water, research on the underlying degradation and transformation mechanisms in plants remains rare. For instance, *V. natans* removed 99.7% of sulfonamides within 13 days [12], *M. aquaticum* eliminated >85% of tetracyclines within 12 weeks [16], and *Ceratophyllum demersum* removed up to 44% of ciprofloxacin from aquaculture effluents [17]. *Egeria densa* achieved 58% ciprofloxacin removal within 96 hours [18]. While these results demonstrate the potential of submerged macrophytes to remove antibiotics, most studies have focused on pollutant reduction efficiency rather than elucidating metabolic pathways or identifying plant-derived antibiotic metabolites, which may exhibit distinct chemical structures and ecotoxicological properties compared to the parent compounds [19]. *H. verticillata* is a widely distributed submerged macrophyte known for its strong biological purification capacity and adaptability to diverse environmental conditions [20]. It can tolerate low light intensity, limited nutrient availability, and a wide range of physical and chemical water conditions, enabling it to outcompete other aquatic plants across broad habitats. These attributes make *H. verticillata* a particularly suitable candidate for phytoremediation of antibiotic-contaminated waters.

Therefore, a comprehensive understanding of antibiotic phytotoxicity, biodegradation, and metabolic transformations in submerged macrophytes is essential to optimize phytoremediation strategies and assess ecological risks of antibiotics. Ciprofloxacin (CIP), a widely used fluoroquinolone, is frequently detected in surface waters with median concentration of 0.164 mg/L and maximum concentration of 6.5 mg/L in freshwater ecosystem, posing considerable ecological risks [21,22]. This study systematically investigated (i) the phytotoxic effects of CIP on the growth and photosynthesis of *H. verticillata*, (ii) antioxidant defense responses including SOD and anthocyanin regulation, (iii) the biodegradation efficiency and metabolic pathways of CIP within plant tissues, and (iv) the associated changes in epiphytic bacterial communities of *H. verticillata*. This study provides new insights into the role of *H. verticillata* in CIP removal and the potential application of submerged macrophytes in phytoremediation of antibiotic-contaminated waters.

## 2. Materials and Methods

### 2.1. Plant Materials and Chemicals

*H. verticillata* used in this experiment was collected in July 2024 from a shallow lake near Hubei University of Technology. *H. verticillata* with green coloration, intact apical tips, and no visible signs of damage or algae infestation were chosen. Plants were carefully washed with deionized water to remove sediment and epiphytes. Prior to experiment, plants were pre-cultured in 10% Hoagland nutrient solution for 2 weeks under controlled conditions (25 ± 1 °C, light intensity 3000 lx, 12 h light/12 h dark cycle) to ensure uniform physiological status. CIP (CIP hydrochloride, ≥97% purity) was purchased from Aladdin (Shanghai, China). The CIP used in this study was in the form of CIP hydrochloride which is highly soluble in water. Stock solutions (1000 mg/L) were prepared in ultrapure water, filtered through 0.22 μm PTFE membranes (Tianjin Jinteng Experiment Equipment Co., Ltd., Tianjin, China), and stored at 4 °C in the dark until use. The filtration process did not cause any significant loss of CIP [23,24,25].

### 2.2. Batch Experiments

#### 2.2.1. Ecotoxicological Effects of CIP on *H. verticillata*

The *H. verticillata* (15 cm in length, fresh weight 1.1 ± 0.1 g) were cultured in a 2 L container with 10% Hoagland solution and anchored with 50 mm glass beads. CIP was added at 0, 1, 2, 5, and 10 mg/L. These concentrations were chosen based on concentrations reported in aqueous environments and other toxicological studies [26]. A CIP-free control was included. All treatments were performed with three independent biological replicates. The plants were incubated in a growth chamber (25 °C, 3000 lx, 12 h light/12 h dark cycle) for 12 days. Evaporation losses were replenished daily with deionized water. At the end of the experiment, plants were harvested for biomass, growth, and biochemical analyses, and culture solutions were collected for antibiotic quantification. Relative growth rate was calculated according to reference [10].

#### 2.2.2. Biodegradation of CIP by *H. verticillata*

To evaluate plant-mediated biodegradation of CIP by *H. verticillata*, pre-cultured plants were trimmed. Two CIP concentrations (1 and 5 mg/L) were tested under three biomass densities in container (fresh weight): low (1.4 g), medium (2.8 g), and high (4.2 g), simulating natural densities of submerged macrophytes in lakes [13]. Blank controls (CIP solution without plants) were included. All treatments were conducted in triplicate. Plants were cultured in a 2 L container with 10% Hoagland solution for 12 days under the same incubation conditions described above.

### 2.3. Assessment of Biochemical Component Changes in H. verticillata

Chlorophyll fluorescence was measured using a portable plant efficiency analyzer (PEA, Hansatech, UK). Leaves were dark-adapted for 15 min before determining the maximum photochemical efficiency of PSII (Fv/Fm) [27]. Chlorophyll and carotenoids were extracted in 95% methanol, and absorbance was measured at 665, 652, and 470 nm. Pigment concentrations were calculated according to reference [11]. SOD activity was determined by nitroblue tetrazolium (NBT) photoreduction [28]. Anthocyanins were extracted in acidified ethanol (1% HCl, *v*/*v*) and quantified at 530 nm using a UV–vis spectrophotometer, the relative concentration of astaxanthin was calculated by the change in absorbance [29].

### 2.4. Determination of CIP Concentrations and CIP Metabolites

#### 2.4.1. CIP Quantification

CIP in culture media was quantified using HPLC (Ultimate 3000, Thermo Fisher Scientific, Waltham, MA, USA) with a C18 column (250 × 4.6 mm, 5 μm) at 40 °C [30]. The mobile phase was methanol/0.04% phosphoric acid buffer with 0.05% triethylamine (70:30, *v*/*v*). The flow rate was 1.0 mL/min, injection volume 10 μL, and UV detection wavelength 278 nm. The retention time of CIP was 5.97 min.

CIP in *H. verticillata* tissues was performed according to reference [31]. At the end of the exposure experiment, leaves and stems of *H. verticillata* were separately collected and freeze-dried for 24 h, followed by grinding into fine powder. Tissue powder was extracted with 5 mL acetonitrile by vortexing for 15 min. The extracts were centrifuged at 8000 r/min for 10 min, and the supernatants were filtered through 0.22 μm organic membranes. The filtrates were analysis using Q Exactive UHPLC-Q Exactive Orbitrap MS (Thermo Fisher Scientific, USA).

#### 2.4.2. Metabolite Identification

CIP metabolites were analyzed using UHPLC-Q Exactive Orbitrap MS (Thermo Fisher Scientific, USA) with a ZORBAX SB-C18 column (2.1 × 50 mm) [6]. Culture samples were centrifuged and extracted three times with ethyl acetate. The extracts were dried over anhydrous Na_2_SO_4_, evaporated under N_2_ and redissolved in methanol. Chromatographic separation was performed with a binary gradient: (A) water with 0.1% formic acid and (B) methanol with 0.1% formic acid, injection volume 1 μL. Mass spectrometry was operated in positive electrospray ionization (ESI^+^) mode at 4.0 kV. Data were acquired in full MS/dd-MS² mode (resolution 70,000), and metabolites were annotated by comparison with the mzCloud and ChemSpider databases using Xcalibur 3.0 software.

### 2.5. DNA Extraction and High Throughout Sequencing

Apical 10 cm segments of *H. verticillata* were transferred into 50 mL centrifuge tubes with 45 mL PBS buffer, shaken for 30 min, ultrasonicated at 40 kHz for 10 min, and shaken again for 10 min. This was repeated three times until leaves regained their natural green color. The washing solution was centrifuged at 8000 rpm for 10 min, and the pellet was stored at −80 °C. Samples were subsequently sent to Shanghai Meiji Biomedical Technology Co., Ltd. (Shanghai, China) for microbial analysis [32].

Total microbial DNA was extracted using the E.Z.N.A.^®^ Soil DNA Kit (Omega Bio-tek, Norcross, GA, USA) according to the manufacturer’s instructions. DNA quality was checked by 1% agarose gel electrophoresis, and concentrations were quantified using a NanoDrop 2000 spectrophotometer. The V3–V4 hypervariable regions of the bacterial 16S rRNA gene were amplified using primers 338F (5′-ACTCCTACGGGAGGCAGCAG-3′) and 806R (5′-GGACTACHVGGGTWTCTAAT-3′) with sample-specific barcodes. PCR amplification was carried out in 20 μL reactions containing 4 μL 5× FastPfu buffer, 2 μL dNTPs (2.5 mM), 0.8 μL each primer (5 μM), 0.4 μL FastPfu polymerase, and 10 ng template DNA. Cycling conditions were: 95 °C for 3 min, followed by 27 cycles of 95 °C for 30 s, 55 °C for 30 s, 72 °C for 30 s, and a final extension at 72 °C for 10 min.

PCR products were purified using a PCR Clean-Up Kit (Yuhua, China) and quantified by Qubit 4.0. Libraries were constructed using the NEXTFLEX Rapid DNA-Seq Kit, and sequencing was performed on the Illumina NovaSeq 6000 platform (Illumina, San Diego, CA, USA) with 2 × 250 bp paired-end reads.

### 2.6. Statistical Analyses

All data were tested for normality and homogeneity of variance prior to analysis. One-way ANOVA was used to assess treatment effects, followed by least significant difference (LSD) and Duncan’s multiple range tests for pairwise comparisons (*p* < 0.05). Statistical analyses were conducted using IBM SPSS Statistics 25.0 (IBM, Armonk, NY, USA). Graphical visualizations were produced using Origin 9.0 (OriginLab, Northampton, MA, USA). Microbial data analyses were carried out on the Majorbio Cloud Platform (https://cloud.majorbio.com, accessed on 10 August 2025).

## 3. Results and Discussion

### 3.1. Effects of CIP the Growth of H. verticillate

**Figure 1:**
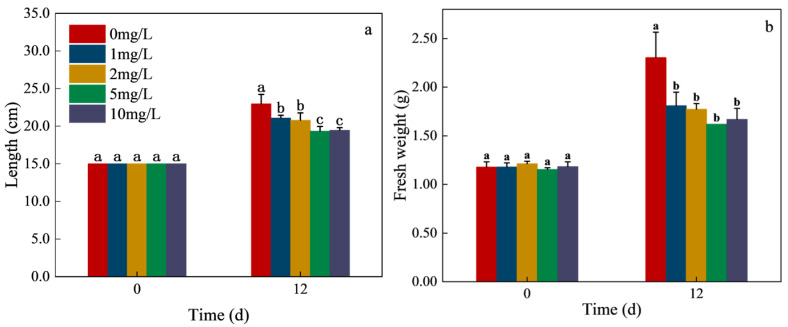
Effects of CIP concentrations on the length (**a**) and fresh weight (**b**) of *H. verticillata* after 12 days of exposure. The concentrations corresponding to different colors in the legends are 0 mg/L (control), 1 mg/L, 2 mg/L, 5 mg/L, and 10 mg/L, respectively. Error bars represent the mean ± standard deviation (*n* = 3). Different letters above the columns indicate significant differences (*p* < 0.05) between the control and experimental groups.

Plant growth is a key endpoint in evaluating the phytotoxicity of CIP, as it reflects the capacity of submerged macrophytes to tolerate and potentially remediate xenobiotics. As shown in Figure 1a,b, the length and fresh weight of *H. verticillata* in the control group increased significantly after 12 days, indicating healthy growth under laboratory conditions. However, CIP exposure markedly suppressed growth. The inhibition of *H. verticillata* length was concentration-dependent, with reductions of 8.22%, 9.48%, 15.82%, and 15.28% at 1, 2, 5, and 10 mg/L CIP, respectively (Figure 1a). Fresh weight exhibited even greater sensitivity, with inhibition rates of 21.42%, 23.01%, 29.66%, and 27.50% at the corresponding concentrations (Figure 1b).

These results demonstrate that CIP exerted stronger inhibitory effects on biomass accumulation than on elongation. This pattern is consistent with previous studies on submerged macrophytes. For example, tetracycline significantly reduced the fresh weight of *Myriophyllum aquaticum* at 10 mg/L, with roots and leaves being more sensitive than stems [11]. Similarly, sulfamethoxazole exposure suppressed leaf and root elongation of *Vallisneria natans*, with biomass showing greater inhibition than morphological indices [12]. The higher sensitivity of biomass to xenobiotics suggests that fresh weight is a more reliable indicator of phytotoxic stress than length growth, likely due to the cumulative impacts of metabolic disruption and photosynthetic inhibition.

### 3.2. Effects of CIP the Photosynthesis of H. verticillata

**Figure 2:**
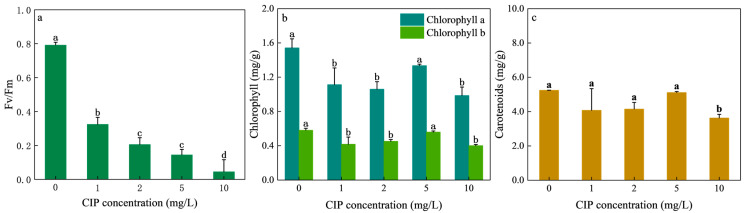
Effects of CIP concentrations on the Fv/Fm (**a**), chlorophyll a and b (**b**), and carotenoids (**c**) of *H. verticillata* after 12 days of exposure. Error bars represent the mean ± standard deviation (*n* = 3). Different letters above the columns indicate significant differences (*p* < 0.05) between the control and experimental groups.

CIP exposure significantly impaired the photosynthesis of *H. verticillata*. As shown in Figure 2a, the maximum photochemical efficiency (Fv/Fm) decreased sharply with increasing CIP concentrations. Compared with the control (0.792), Fv/Fm values dropped to 0.325, 0.206, 0.145, and 0.045 at 1, 2, 5, and 10 mg/L CIP, representing decreases of 59.01%, 73.99%, 81.73%, and 94.32%, respectively. The effects of CIP on the chlorophyll a, chlorophyll b content of *H. verticillata* are shown in Figure 2b. Relative to the control, chlorophyll a of *H. verticillata* decreased by 27.85%, 31.33%, 13.41%, and 36.10% at 1, 2, 5, and 10 mg/L, while chlorophyll b decreased by 28.17%, 22.33%, 3.84%, and 31.20%. Carotenoid levels were comparatively less affected (Figure 2c), showing only slight reductions under CIP exposure except at the highest concentration.

Chlorophyll fluorescence has been widely used to assess plant responses to stress [27]. Fv/Fm, an indicator of PSII efficiency to ROS accumulation [33], revealed severe photoinhibition in *H. verticillata*. Generally speaking, Fv/Fm values lower than 0.2 indicate severe PSII damage, highlighting strong, concentration-dependent photoinhibition [34]. The observed declines demonstrate that CIP disrupted PSII reaction centers and impaired electron transport, thereby reducing light energy conversion. Similar inhibition of PSII was observed in *V. natans* exposed to oxytetracycline and sulfadiazine [13], supporting that fluoroquinolones can significantly impair the photosynthetic machinery of submerged plants.

Chlorophylls and carotenoids are fundamental pigments for light harvesting, energy absorption, and photosynthetic metabolism. The strong reduction in chlorophyll a and b content observed here suggests that CIP interfered with chloroplast structure and chlorophyll biosynthesis, while also accelerating degradation via chlorophyllase activity [35,36]. Comparable chlorophyll reductions were reported in *V. natans* under sulfonamide stress [12], where photosynthetic inhibition translated into growth suppression. Notably, leaf yellowing observed in *H. verticillata* at 10 mg/L CIP corresponds with severe chlorophyll loss, serving as a visible indicator of phytotoxicity.

Carotenoids, in contrast, are key non-enzymatic antioxidants that scavenge ROS and protect chlorophyll under stress [37]. The relatively stable carotenoid levels in *H. verticillata* suggest a protective defense mechanism, consistent with previous findings where carotenoid stability was maintained under phenolic stress [38,39]. However, under 10 mg/L CIP, the marked decline in carotenoids indicates that antioxidant defenses were overwhelmed, leading to irreversible metabolic impairment.

### 3.3. Effects of Oxidative Stress by CIP on H. verticillate

**Figure 3:**
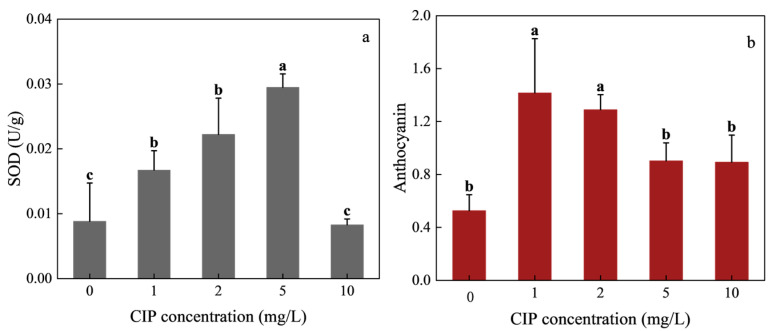
Effects of CIP concentrations on SOD activity (**a**) and anthocyanin (**b**) in *H. verticillata* after 12 days of exposure. Error bars represent the mean ± standard deviation (*n* = 3). Different letters above the columns indicate significant differences (*p* < 0.05) between the control and experimental groups.

Superoxide dismutase (SOD) activity exhibited a clear biphasic response to CIP exposure (Figure 3a). At 1, 2, and 5 mg/L CIP, SOD activity increased by 89.19%, 151.56%, and 233.69% relative to the control, peaking at 3.3-fold the control value under 5 mg/L exposure. However, at 10 mg/L, SOD activity dropped by 6.4% compared with the control, showing no significant difference (*p* > 0.05). Anthocyanin content followed a similar trend (Figure 3b). After 12 days of cultivation, all CIP-treated groups contained significantly more anthocyanins than the control. Increases were 168.99%, 144.94%, 71.52%, and 69.62% at 1, 2, 5, and 10 mg/L, respectively. The strongest accumulation occurred under 1–2 mg/L CIP exposure, while higher concentrations dampened this stimulatory effect.

SOD is the first line antioxidant enzyme in plants, catalyzing the dismutation of superoxide radicals into hydrogen peroxide and oxygen, thereby protecting membranes and organelles from oxidative damage. The sharp increase in SOD activity at 1–5 mg/L CIP suggests that *H. verticillata* initiated an active defense against CIP-induced reactive oxygen species (ROS). Such activation of antioxidant defenses has also been reported for *Vallisneria natans*, where low-level CIP exposure stimulated SOD activity but higher concentrations suppressed it [26]. The decline at 10 mg/L in *H. verticillata* indicates that ROS generation exceeded the enzymatic detoxification capacity, leading to disruption of antioxidant regulation and subsequent oxidative damage [40].

Anthocyanins, non-enzymatic antioxidants belonging to the flavonoid family, also responded in a biphasic pattern. At low CIP concentrations, enhanced ROS signaling likely activated transcriptional pathways for anthocyanin biosynthesis [41,42], providing additional protection against oxidative stress. However, at higher concentrations, anthocyanin accumulation was suppressed. This may reflect accelerated degradation of anthocyanins under severe oxidative conditions or inhibition of biosynthetic pathways caused by widespread metabolic impairment. Similar trends have been observed in *Chlorella vulgaris* exposed to cefazolin sodium, where anthocyanins first increased then declined at higher doses [43].

In general, antioxidant activity increases under relatively low levels of environmental stress but declines under more severe conditions due to irreversible cellular damage. The simultaneous increases in SOD and anthocyanin activities observed in this study indicate that both enzymatic and non-enzymatic antioxidants act synergistically to mitigate stress-induced damage. This hormesis-like response suggests that low concentrations of CIP stimulate antioxidant defenses, which holds significant implications for understanding plant tolerance mechanisms. Collectively, these results reveal a hormesis response of *H. verticillata* to CIP-induced oxidative stress: low-to-moderate CIP concentrations enhance antioxidant defenses, whereas excessive stress overwhelms the system, leading to antioxidant suppression. This different response underscores the importance of oxidative stress modulation in determining plant tolerance thresholds to antibiotics in aquatic environments.

### 3.4. Biodegradation Efficiency and Pathway of CIP by H. verticillata

**Figure 4:**
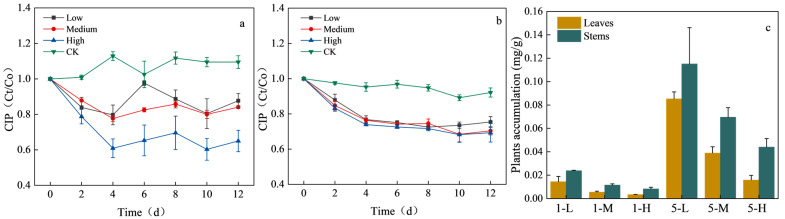
Biodegradation of CIP at different concentrations by *H. verticillata* ((**a**): 1 mg/L, (**b**): 5 mg/L, (**c**): CIP accumulation in stems and leaves). The biomass densities of *H. verticillata* corresponding to different digital suffixes in the legends are 0 g (CK), 1.4 g (low), 2.8 g (medium), and 4.2 g (high), respectively. Error bars represent the standard error of the mean (*n* = 3).

CIP concentrations in the aqueous phase during the 12-day experiment are shown in Figure 4. In the control groups (without plants), CIP concentrations remained nearly constant at both 1 mg/L and 5 mg/L, confirming negligible abiotic loss. By contrast, treatments containing *H. verticillata* exhibited significant concentration declines. At 1 mg/L, removal efficiencies reached 12.33%, 12.31%, and 36.96% under low (L), medium (M), and high (H) planting densities, respectively. At 5 mg/L, corresponding removal efficiencies were 25.62%, 29.60%, and 30.69%. These results indicate that removal was biomass-dependent, with higher plant densities achieving greater removal, particularly under the 1 mg/L condition (Figure 4a). At 5 mg/L (Figure 4b), removal increased with biomass but tended to plateau between medium and high densities, suggesting a potential saturation of uptake or metabolic capacity.

CIP accumulation in plant tissues at the end of the experiment is presented in Figure 4c. In the 1 mg/L treatment, leaf accumulation reached 0.014, 0.006, and 0.003 mg/g in the L, M, and H groups, respectively, while stem accumulation was 0.024, 0.012, and 0.008 mg/g. In the 5 mg/L treatment, leaf accumulation increased to 0.085, 0.039, and 0.016 mg/g, while stem accumulation reached 0.115, 0.069, and 0.044 mg/g. Across treatments, stems consistently accumulated higher levels than leaves, and accumulation was substantially greater under 5 mg/L than under 1 mg/L. These findings suggest that *H. verticillata* contributes to CIP removal through a combination of uptake, adsorption, and potential metabolic degradation. The biomass dependence indicates that greater plant surface area and metabolic activity enhance removal efficiency. Notably, stems accumulated more CIP than leaves, likely due to differences in tissue composition and transport capacity, which may favor storage in structural tissues. Similar preferential accumulation in stems has been reported for sulfonamides in *Vallisneria natans* [12]. Moreover, many antibiotics can be absorbed and accumulated in various plant tissues. For instance, *Myriophyllum aquaticum* removed tetracycline from water, accumulated in roots, stems, and leaves [11]. Likewise, CIP has been reported to accumulate in the roots of *Eichhornia crassipes*. Sufficient lipophilicity allows CIP to cross the lipid bilayer of plant cell membranes, while adequate hydrophilicity facilitates its translocation through cell sap [44]. These results indicate that antibiotic accumulation in aquatic plants is not limited to passive adsorption but also involves active transportation and partition. Compared with advanced oxidation processes (AOPs), the removal capacity of *H. verticillata* remains limited. However, since antibiotics typically occur in aquatic environments at concentrations ranging from ng/L to μg/L, the performance of *H. verticillata* in removing CIP under such environmentally relevant concentrations and at larger scales remains promising and needs further investigation.

**Figure 5:**
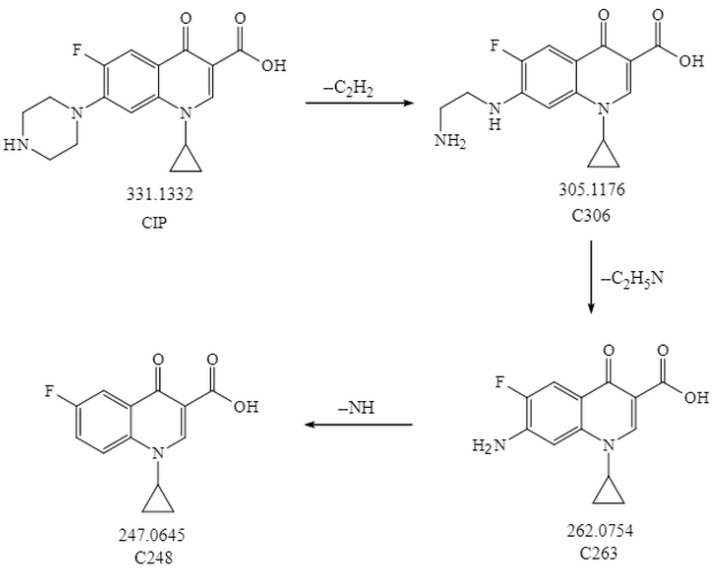
Proposed biodegradation pathway for CIP by *H. verticillata*. The arrows linking two chemicals indicate putative processes. The numbers represent the exact mass of the chemical compounds.

To elucidate the mechanism of CIP biodegradation in the *H. verticillata* cultivation system, degradation intermediates were identified using UHPLC-MS/MS. A total of three metabolites C306, C263, and C248 were detected, with their molecular formulas, molecular weights, and proposed structures summarized in Table 1. Details on their MS spectra are summarized in the Supplementary Material (Figures S1–S4). Based on these results, a possible biodegradation pathway of CIP is proposed in Figure 5. Specifically, CIP undergoes partial ring-opening to yield C306. Subsequent removal of the entire piperazine ring produces C263, which is further deaminated to form C248.

In this study, analysis of intermediate products supports the hypothesis that *H. verticillata* contributes to active biodegradation of CIP. The proposed degradation pathway involves sequential N-deethylation to form C306, piperazine-ring cleavage to produce C263, and deamination yielding C248. Such reactions are consistent with the chemical reactivity of fluoroquinolones, as the piperazine moiety is particularly prone to substitution or decomposition via deethylation and deamination [45]. Indeed, C306 has been identified as a CIP metabolite in nitrifying biofilm systems [45], and also detected during CIP degradation by *Gloeophyllum striatum* [46,47]. Similarly, C263, lacking the piperazine moiety at the C7 position, was observed in the roots of *Vetiveria zizanioides* exposed to CIP [31]. These findings confirm that the transformation products observed in *H. verticillata* align with well-documented biodegradation routes of fluoroquinolones in both microbial and plant systems.

### 3.5. Effects of CIP on the Epiphytic Bacterial Communities of H. verticillata

**Figure 6:**
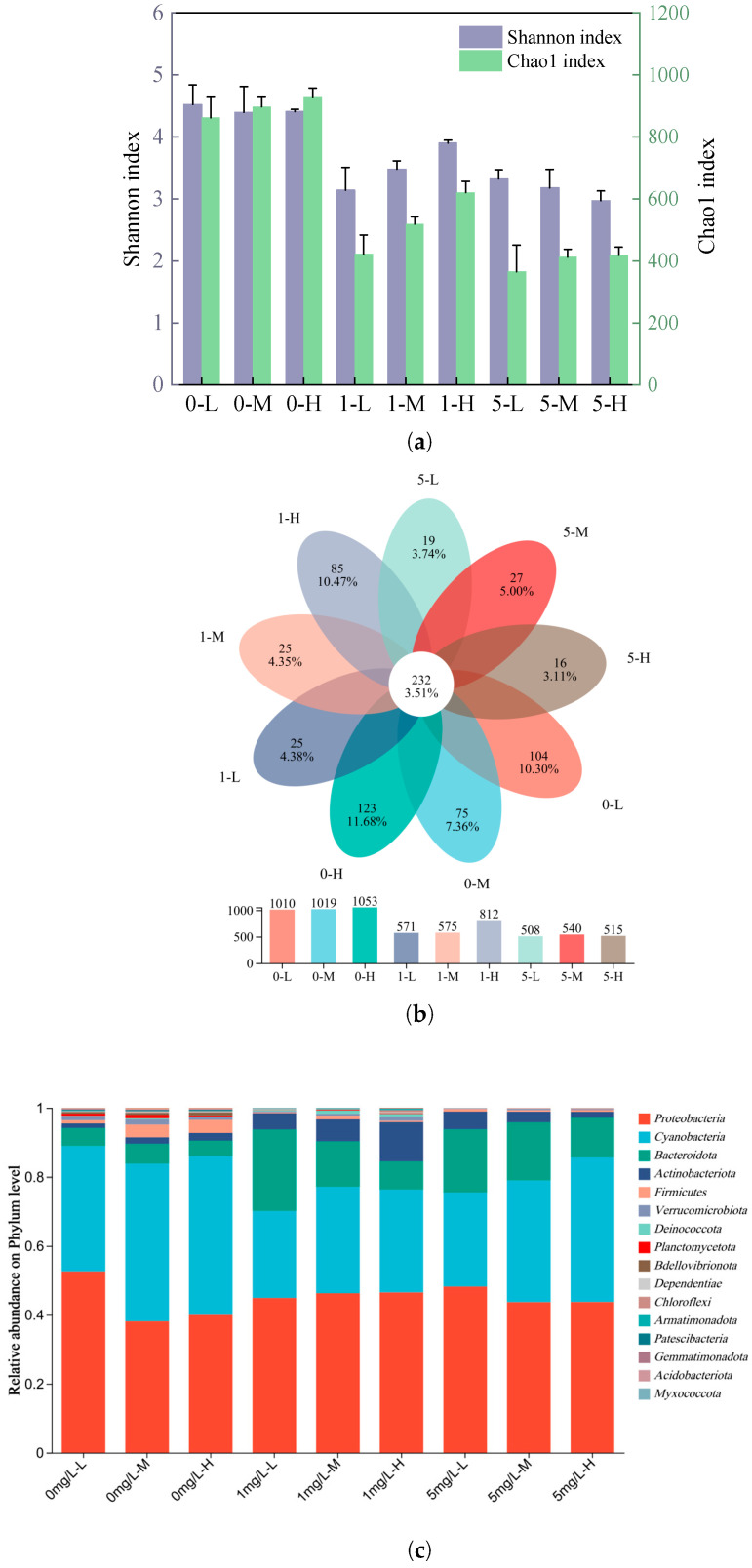

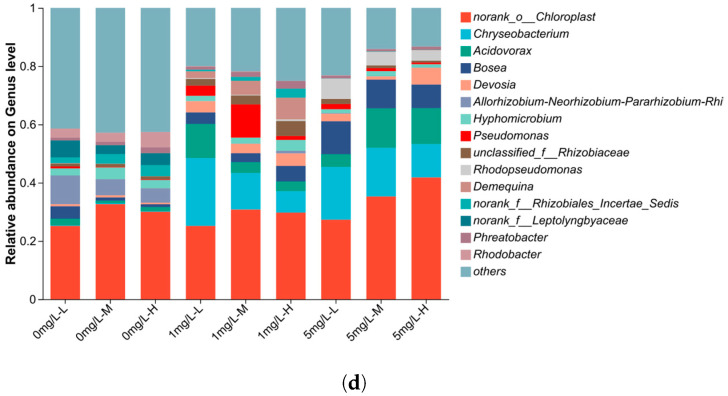
Effects of CIP on the epiphytic bacterial communities of *H. verticillata*. (**a**) Shannon and Chao1 Indices, (**b**) Venn diagram, (**c**) microbial population dynamics at phylum level, (**d**) microbial population dynamics at genus level.

To investigate the impact of CIP on bacterial communities associated with biodegradation, 16S rRNA sequencing was conducted. Alpha diversity indices (Shannon and Chao1) showed that CIP exposure significantly reduced both bacterial richness and diversity compared with controls (Figure 6a), indicating that CIP stress simplified the structural complexity of epiphytic microbiomes. A Venn diagram further revealed that CIP markedly reduced the number of unique OTUs (Figure 6b), suggesting a decline in microbial richness and overlap. Such losses are consistent with the selective pressure of antibiotics, which favor resistant populations while suppressing sensitive taxa [48,49].

At the phylum level (Figure 6c), *Proteobacteria* and *Cyanobacteria* were the dominant groups, accounting for more than 70% of the communities, followed by *Bacteroidota*, *Actinobacteria*, and *Firmicutes*. CIP exposure increased *Actinobacteria* and *Bacteroidota* while reducing *Firmicutes*, reflecting differential resistance of microorganism. The mutations in the DNA gyrase (gyrA/B) of *Actinobacteria* can reduce CIP binding affinity [50], while *Bacteroidota* utilize efflux pumps *AcrAB-TolC* to expel antibiotics [51]. Consistent with our results, CIP exposure has been shown to enhance the abundance of *Actinobacteria* in biofilms associated with *Vallisneria natans* [52]. Conversely, the decline of *Firmicutes* may be explained by their selective inhibition under fluoroquinolone stress [53].

At the genus level (Figure 6d), *norank_o__Chloroplast*, *Chryseobacterium*, *Acidovorax*, *Bosea*, and *Devosia* were particularly abundant. *Norank_o__Chloroplast* sequences, derived from chloroplasts, represent photosynthetic organisms capable of oxygen release during daytime, which may interfere with denitrification processes [54]. Their abundance increased with plant biomass, likely reflecting enhanced epiphytic colonization. *Chryseobacterium* are Gram-negative rods with intrinsic antibiotic resistance [55]. *Acidovorax*, also Gram-negative, have capacity to degrade polycyclic aromatic hydrocarbons (PAH) phenanthrene [56]. *Bosea* can produce β-lactamases and L,D-transpeptidases or homologous enzymes that enable the degradation of antibiotics such as amoxicillin [57]. *Devosia*, belonging to the *Proteobacteria*, are root-colonizing bacteria that can directly promote plant growth [58], and *Proteobacteria* were the main microbes to remediate piperazine contained wastewater which similar to the degradation pathway of CIP [59]. The increased abundance of these genera under CIP exposure indicates both antibiotic resistance and potential roles in antibiotic biodegradation. Collectively, these results demonstrate that CIP exposure alters the microbial ecology of *H. verticillata* associated communities, reducing the diversity while selectively enriching resistant genera. Such community shifts may synergistically contribute to the observed biodegradation of CIP through plant microbe interactions.

## 4. Conclusions

This study demonstrated that CIP exerts phytotoxicity on *H. verticillata* through inhibited growth, reduced photosynthetic efficiency, and oxidative stress imbalance. Nevertheless, *H. verticillata* contributes to CIP removal in aquatic systems. Three degradation intermediates (C306, C263, and C248) were identified, and the possible biodegradation pathway was proposed, involving de-ethylation, piperazine ring cleavage, and deamination. Furthermore, CIP exposure alters the epiphytic microbial community, favoring antibiotic-resistant genera while reducing overall bacterial diversity. These findings indicate that *H. verticillata* is a potential agent for CIP removal from aquatic environments in phytoremediation. Future research should focus on long-term exposure experiments at environmentally relevant concentrations to fully evaluate the practical applicability of *H. verticillata* in phytoremediation.

## Figures and Tables

**Table 1:** The MS spectra information of CIP and the proposed structures of its metabolites.

Compounds	Molecular Formula	Exact Mass	Retention Time	Experimental [M + H]^+^	Error (ppm)	Proposed Structure
CIP	C_17_H_18_FN_3_O_3_	331.1332	4.46	332.1405	−0.079	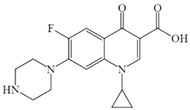
C306	C_15_H_16_FN_3_O_3_	305.1176	4.20	306.1253	0.013	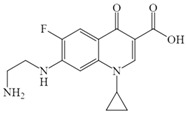
C263	C_13_H_11_FN_2_O_3_	262.0754	5.76	263.0827	0.202	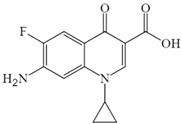
C248	C_13_H_10_FNO_3_	247.0645	5.73	248.0718	0.250	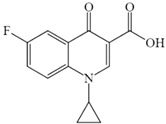

## Data Availability

The original contributions presented in this study are included in the article/Supplementary Material. Further inquiries can be directed to the corresponding author.

## Supplementary Materials

The following supporting information can be downloaded at: https://www.mdpi.com/article/10.3390/toxics13100882/s1, Figure S1: The MS/MS spectra of ciprofloxacin; Figure S2: The MS/MS spectra of biodegradation product C306; Figure S3: The MS/MS spectra of biodegradation product C263; Figure S4: The MS/MS spectra of biodegradation product C248.

## References

[1] Browne A.J., Chipeta M.G., Haines-Woodhouse G., Kumaran E.P.A., Hamadani B.H.K., Zaraa S., Henry N.J., Deshpande A., Reiner R.C., Day N.P.J. (2021). Global Antibiotic Consumption and Usage in Humans, 2000–18: A Spatial Modelling Study. Lancet Planet Health.

[2] Klein E.Y., Impalli I., Poleon S., Denoel P., Cipriano M., Van Boeckel T.P., Pecetta S., Bloom D.E., Nandi A. (2024). Global Trends in Antibiotic Consumption during 2016–2023 and Future Projections through 2030. Proc. Natl. Acad. Sci. USA.

[3] Mulchandani R., Wang Y., Gilbert M., Van Boeckel T.P. (2023). Global Trends in Antimicrobial Use in Food-Producing Animals: 2020 to 2030. PLOS Glob. Public Health.

[4] Kovalakova P., Cizmas L., McDonald T.J., Marsalek B., Feng M., Sharma V.K. (2020). Occurrence and Toxicity of Antibiotics in the Aquatic Environment: A Review. Chemosphere.

[5] Ren Z., Xu H., Wang Y., Li Y., Han S., Ren J. (2021). Combined Toxicity Characteristics and Regulation of Residual Quinolone Antibiotics in Water Environment. Chemosphere.

[6] Wan L., Wu Y., Zhang Y., Zhang W. (2022). Toxicity, Biodegradation of Moxifloxacin and Gatifloxacin on *Chlamydomonas Reinhardtii* and Their Metabolic Fate. Ecotoxicol. Environ. Saf..

[7] Zhao Z., Qin Z., Xia L., Zhang D., Hussain J. (2018). Dissipation Characteristics of Pyrene and Ecological Contribution of Submerged Macrophytes and Their Biofilms-Leaves in Constructed Wetland. Bioresour. Technol..

[8] Zhang H., Luo X., Li Q., Huang S., Wang N., Zhang D., Zhang J., Zheng Z. (2020). Response of the Submerged Macrophytes *Vallisneria natans* to Snails at Different Densities. Ecotoxicol. Environ. Saf..

[9] Rocha D.C., Da Silva Rocha C., Tavares D.S., De Morais Calado S.L., Gomes M.P. (2021). Veterinary Antibiotics and Plant Physiology: An Overview. Sci. Total Environ..

[10] Zhang L., Zhang Y., Liu L. (2019). Effect of Submerged Macrophytes *Vallisneria spiralis* L. on Restoring the Sediment Contaminated by Enrofloxacin in Aquaculture Ponds. Ecol. Eng..

[11] Guo X., Liu M., Zhong H., Li P., Zhang C., Wei D., Zhao T. (2020). Responses of the Growth and Physiological Characteristics of *Myriophyllum aquaticum* to Coexisting Tetracyclines and Copper in Constructed Wetland Microcosms. Environ. Pollut..

[12] Zhu L., Xu H., Xiao W., Lu J., Lu D., Chen X., Zheng X., Jeppesen E., Zhang W., Wang L. (2020). Ecotoxicological Effects of Sulfonamide on and Its Removal by the Submerged Plant *Vallisneria natans* (Lour.) Hara. Water Res..

[13] Zhang H., Ge Z., Li Y., Huang S., Zhang J., Zheng Z. (2022). Response of Submerged Macrophytes and Leaf Biofilms to Different Concentrations of Oxytetracycline and Sulfadiazine. Chemosphere.

[14] Dai Y., Tang H., Chang J., Wu Z., Liang W. (2014). What’s Better, *Ceratophyllum demersum* L. or *Myriophyllum verticillatum* L., Individual or Combined?. Ecol. Eng..

[15] Gomes M.P., Gonçalves C.A., De Brito J.C.M., Souza A.M., Da Silva Cruz F.V., Bicalho E.M., Figueredo C.C., Garcia Q.S. (2017). Ciprofloxacin Induces Oxidative Stress in Duckweed (*Lemna minor* L.): Implications for Energy Metabolism and Antibiotic-Uptake Ability. J. Hazard. Mater..

[16] Guo X., Liu M.M., Zhong H., Li P., Zhang C.J., Wei D., Zhao T.K. (2020). Potential of *Myriophyllum aquaticum* for Phytoremediation of Water Contaminated with Tetracycline Antibiotics and Copper. J. Environ. Manag..

[17] Hoang T.T.T., Tu L.T.C., Le N.P., Dao Q.P., Trinh P.H. (2012). Fate of Fluoroquinolone Antibiotics in Vietnamese Coastal Wetland Ecosystem. Wetl. Ecol. Manag..

[18] Kitamura R.S.A., Brito J.C.M., Silva De Assis H.C., Gomes M.P. (2023). Physiological Responses and Phytoremediation Capacity of Floating and Submerged Aquatic Macrophytes Exposed to Ciprofloxacin. Environ. Sci. Pollut. Res..

[19] Löffler P., Escher B.I., Baduel C., Virta M.P., Lai F.Y. (2023). Antimicrobial Transformation Products in the Aquatic Environment: Global Occurrence, Ecotoxicological Risks, and Potential of Antibiotic Resistance. Environ. Sci. Technol..

[20] Shrivastava M., Srivastava S. (2021). Application and Research Progress of *Hydrilla verticillata* in Ecological Restoration of Water Contaminated with Metals and Metalloids. Environ. Chall..

[21] Liu X., Lu S., Guo W., Xi B., Wang W. (2018). Antibiotics in the Aquatic Environments: A Review of Lakes, China. Sci. Total Environ..

[22] Hughes S.R., Kay P., Brown L.E. (2013). Global Synthesis and Critical Evaluation of Pharmaceutical Data Sets Collected from River Systems. Environ. Sci. Technol..

[23] Dong W., Yu X., Wang J., Sui Q. (2022). Is It the Appropriate Syringe Filter? The Loss of PPCPs during Filtration by Syringe Filter. Water Emerg. Contam. Nanoplastics.

[24] Hayri-Senel T., Kahraman E., Sezer S., Erdol-Aydin N., Nasun-Saygili G. (2024). Photocatalytic Degradation of Ciprofloxacin from Water with Waste Polystyrene and TiO_2_ Composites. Heliyon.

[25] Xu Q., Li H., Li S., Li Z., Chen S., Liang Y., Li Y., Li J., Yuan M. (2025). Impact of Microplastics on Ciprofloxacin Adsorption Dynamics and Mechanisms in Soil. Toxics.

[26] Fan P., Liu C., Ke Z., Zhou W., Wu Z. (2022). Growth and Physiological Responses in a Submerged Clonal Aquatic Plant and Multiple-Endpoint Assessment under Prolonged Exposure to Ciprofloxacin. Ecotoxicol. Environ. Saf..

[27] Arief M.A.A., Kim H., Kurniawan H., Nugroho A.P., Kim T., Cho B.-K. (2023). Chlorophyll Fluorescence Imaging for Early Detection of Drought and Heat Stress in Strawberry Plants. Plants.

[28] Wan L., Zhou Y., Huang R., Jiao Y., Gao J. (2024). Toxicity of Moxifloxacin on the Growth, Photosynthesis, Antioxidant System, and Metabolism of *Microcystis aeruginosa* at Different Phosphorus Levels. Toxics.

[29] Pirie A., Mullins M.G. (1976). Changes in Anthocyanin and Phenolics Content of Grapevine Leaf and Fruit Tissues Treated with Sucrose, Nitrate, and Abscisic Acid. Plant Physiol..

[30] Yan Y., Pengmao Y., Xu X., Zhang L., Wang G., Jin Q., Chen L. (2020). Migration of Antibiotic Ciprofloxacin during Phytoremediation of Contaminated Water and Identification of Transformation Products. Aquat. Toxicol..

[31] Panja S., Sarkar D., Li K., Datta R. (2019). Uptake and Transformation of Ciprofloxacin by Vetiver Grass (*Chrysopogon zizanioides*). Int. Biodeterior. Biodegrad..

[32] Zhao X., Wang Y., Cai W., Yang M., Zhong X., Guo Z., Shan C. (2020). High-Throughput Sequencing-Based Analysis of Microbial Diversity in Rice Wine Koji from Different Areas. Curr. Microbiol..

[33] Gomes M.P., Le Manac’h S.G., Hénault-Ethier L., Labrecque M., Lucotte M., Juneau P. (2017). Glyphosate-Dependent Inhibition of Photosynthesis in Willow. Front. Plant Sci..

[34] Li S., Yang W., Yang T., Chen Y., Ni W. (2015). Effects of Cadmium Stress on Leaf Chlorophyll Fluorescence and Photosynthesis of *Elsholtzia argyi*—A Cadmium Accumulating Plant. Int. J. Phytoremediation.

[35] Parida A., Das A.B., Das P. (2002). NaCl Stress Causes Changes in Photosynthetic Pigments, Proteins, and Other Metabolic Components in the Leaves of a True Mangrove, *Bruguiera parviflora*, in Hydroponic Cultures. J. Plant Biol..

[36] Wang C., Zhang S., Wang P., Hou J., Qian J., Ao Y., Lu J., Li L. (2011). Salicylic Acid Involved in the Regulation of Nutrient Elements Uptake and Oxidative Stress in *Vallisneria natans* (Lour.) Hara under Pb Stress. Chemosphere.

[37] Peng J., Guo J., Lei Y., Mo J., Sun H., Song J. (2021). Integrative Analyses of Transcriptomics and Metabolomics in *Raphidocelis subcapitata* Treated with Clarithromycin. Chemosphere.

[38] Eggink L.L., LoBrutto R., Brune D.C., Brusslan J., Yamasato A., Tanaka A., Hoober J.K. (2004). Synthesis of Chlorophyll b: Localization of Chlorophyllide Aoxygenase and Discovery of a Stable Radical in the Catalytic Subunit. BMC Plant Biol..

[39] Chang G., Yue B., Gao T., Yan W., Pan G. (2020). Phytoremediation of Phenol by *Hydrilla verticillata* (L.f.) Royle and Associated Effects on Physiological Parameters. J. Hazard. Mater..

[40] Aderemi A.O., Novais S.C., Lemos M.F.L., Alves L.M., Hunter C., Pahl O. (2018). Oxidative Stress Responses and Cellular Energy Allocation Changes in Microalgae Following Exposure to Widely Used Human Antibiotics. Aquat. Toxicol..

[41] Naing A.H., Kim C.K. (2021). Abiotic Stress-induced Anthocyanins in Plants: Their Role in Tolerance to Abiotic Stresses. Physiol. Plant..

[42] Tena N., Martín J., Asuero A.G. (2020). State of the Art of Anthocyanins: Antioxidant Activity, Sources, Bioavailability, and Therapeutic Effect in Human Health. Antioxidants.

[43] Rezaee A., Kosari-Nasab M., Movafeghi A. (2023). Cellular Toxicity of Cefazolin Sodium to the Green Microalga *Chlorella vulgaris*: Evaluation of Biological Responses. Biologia.

[44] Yan Y., Xu X., Shi C., Yan W., Zhang L., Wang G. (2019). Ecotoxicological Effects and Accumulation of Ciprofloxacin in *Eichhornia crassipes* under Hydroponic Conditions. Environ. Sci. Pollut. Res..

[45] Xu Y., Gu Y., Peng L., Wang N., Chen S., Liang C., Liu Y., Ni B.-J. (2023). Unravelling Ciprofloxacin Removal in a Nitrifying Moving Bed Biofilm Reactor: Biodegradation Mechanisms and Pathways. Chemosphere.

[46] Vasconcelos T.G., Henriques D.M., König A., Martins A.F., Kümmerer K. (2009). Photo-Degradation of the Antimicrobial Ciprofloxacin at High pH: Identification and Biodegradability Assessment of the Primary by-Products. Chemosphere.

[47] Wetzstein H.-G., Stadler M., Tichy H.-V., Dalhoff A., Karl W. (1999). Degradation of Ciprofloxacin by Basidiomycetes and Identification of Metabolites Generated by the Brown Rot Fungus *Gloeophyllum Striatum*. Appl. Environ. Microbiol..

[48] Zhang L., Zhang C., Lian K., Liu C. (2021). Effects of Chronic Exposure of Antibiotics on Microbial Community Structure and Functions in Hyporheic Zone Sediments. J. Hazard. Mater..

[49] Li D., Qi R., Yang M., Zhang Y., Yu T. (2011). Bacterial Community Characteristics under Long-Term Antibiotic Selection Pressures. Water Res..

[50] Drlica K., Malik M. (2003). Fluoroquinolones: Action and Resistance. Curr. Top. Med. Chem..

[51] Piddock L.J.V. (2006). Multidrug-Resistance Efflux Pumps ? Not Just for Resistance. Nat. Rev. Microbiol..

[52] Ohore O.E., Zhang S., Guo S., Addo F.G., Manirakiza B., Zhang W. (2021). Ciprofloxacin Increased Abundance of Antibiotic Resistance Genes and Shaped Microbial Community in Epiphytic Biofilm on *Vallisneria spiralis* in Mesocosmic Wetland. Bioresour. Technol..

[53] Cytryn E. (2013). The Soil Resistome: The Anthropogenic, the Native, and the Unknown. Soil Biol. Biochem..

[54] Zhang S., Zhong Q., Jiang Y., Li M., Xia S. (2021). Temperature-Induced Difference in Microbial Characterizations Accounts for the Fluctuation of Sequencing Batch Biofilm Reactor Performance. Biodegradation.

[55] Kirby J.T., Sader H.S., Walsh T.R., Jones R.N. (2004). Antimicrobial Susceptibility and Epidemiology of a Worldwide Collection of *Chryseobacterium* Spp.: Report from the SENTRY Antimicrobial Surveillance Program (1997–2001). J. Clin. Microbiol..

[56] Singleton D.R., Lee J., Dickey A.N., Stroud A., Scholl E.H., Wright F.A., Aitken M.D. (2018). Polyphasic Characterization of Four Soil-Derived Phenanthrene-Degrading Acidovorax Strains and Proposal of *Acidovorax carolinensis* Sp. Nov. Syst. Appl. Microbiol..

[57] Yan L., Yan N., Gao X.-Y., Liu Y., Liu Z.-P. (2022). Degradation of Amoxicillin by Newly Isolated *Bosea sp.* Ads-6. Sci. Total Environ..

[58] Zhang C., Van Der Heijden M.G.A., Dodds B.K., Nguyen T.B., Spooren J., Valzano-Held A., Cosme M., Berendsen R.L. (2024). A Tripartite Bacterial-Fungal-Plant Symbiosis in the Mycorrhiza-Shaped Microbiome Drives Plant Growth and Mycorrhization. Microbiome.

[59] Zhao L., Pan J., Ding Y., Cai S., Cai T., Chen L., Ji X.-M. (2023). Coupling Continuous Poly(3-Hydroxybutyrate) Synthesis with Piperazine-Contained Wastewater Treatment: Fermentation Performance and Microbial Contamination Deciphering. Int. J. Biol. Macromol..

