# Supplementary material for: Phytotoxic Effects and Microbial Responses to Ciprofloxacin and Its Removal by Hydrilla verticillata
Source: Toxics. 2025 Oct 16;13(10):882. doi: 10.3390/toxics13100882 (PMC12567789; doi:10.3390/toxics13100882)
Supplement: Supplementary file 1 [file toxics-13-00882-s001.zip › toxics-3880746-supplementary.pdf]

Supplementary Materials

Phytotoxic Effects and Microbial Responses to Ciprofloxacin and Its Removal by *Hydrilla verticillata*

Linzhi Lu [1,†], Rong Huang [1, †], Liang Wan [1,2,3,*], Guijia Li [1], Zhenhao Xu [1] and Jiahao Guo [1]

1 *Hubei Key Laboratory of Environmental Geotechnology and Ecological Remediation for Lake & River, School of Civil Engineering, Architecture and Environment, Hubei University of Technology, Wuhan 430068, China*

2 *Key Laboratory of Intelligent Health Perception and Ecological Restoration of Rivers and Lakes, Ministry of Education, Hubei University of Technology, Wuhan 430068, China*

3 *Innovation Demonstration Base of Ecological Environment Geotechnical and Ecological Restoration of Rivers and Lakes, Hubei University of Technology, Wuhan 430068, China*

† These authors contributed equally to this work.

* Correspondence: liangwan@hbut.edu.cn or liangw26@outlook.com

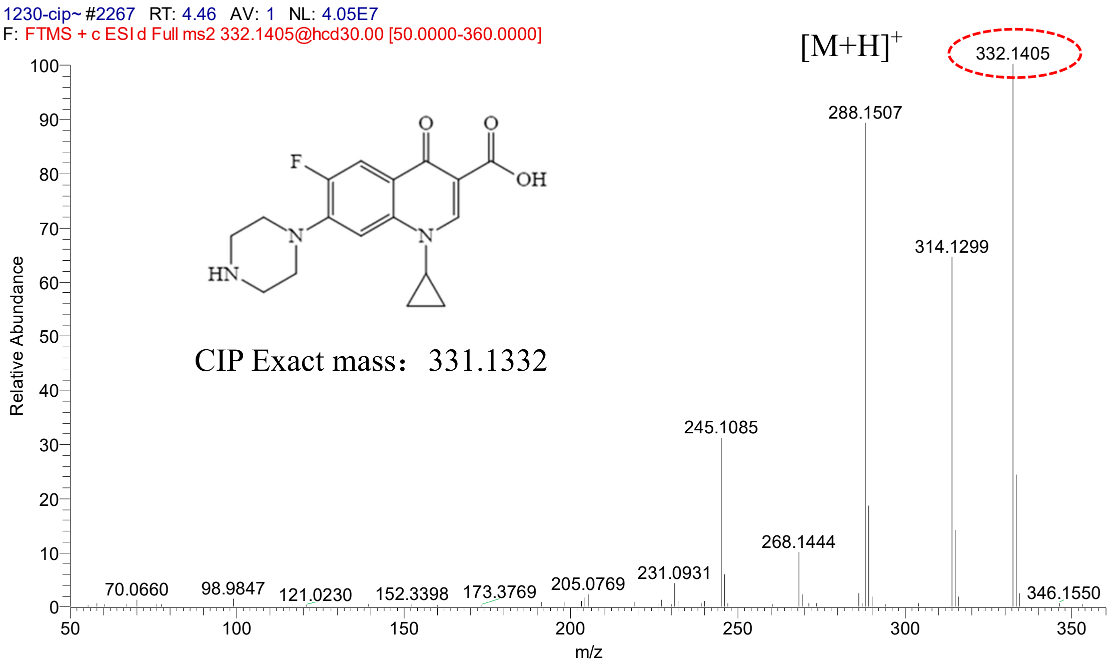

Figure S1 The MS/MS spectra of ciprofloxacin

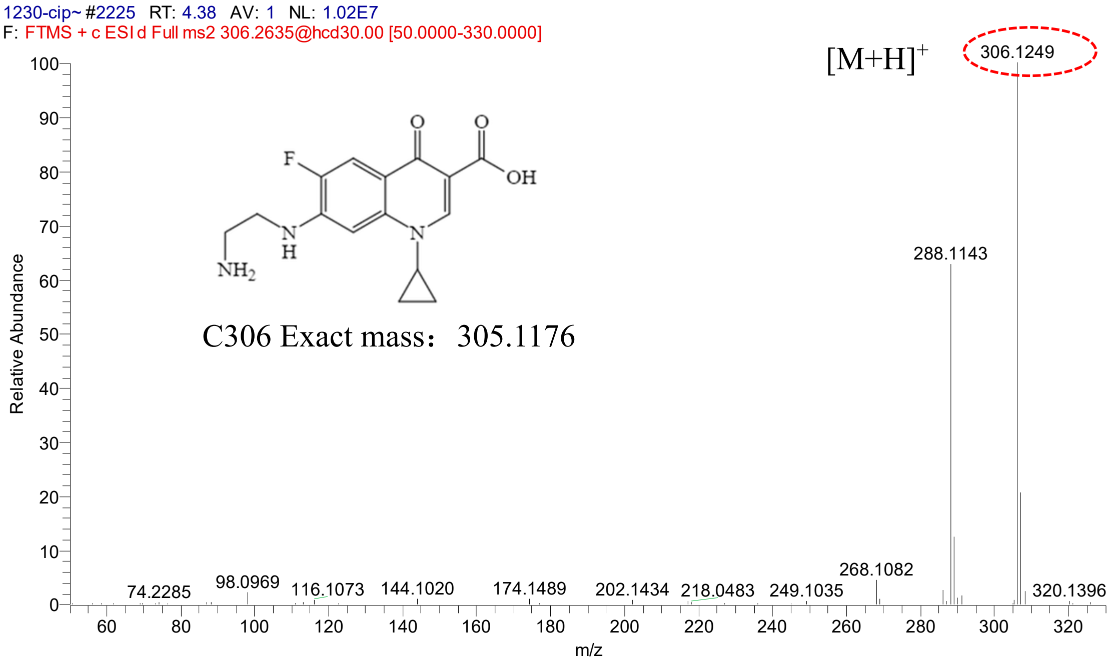

Figure S2 The MS/MS spectra of biodegradation product C306

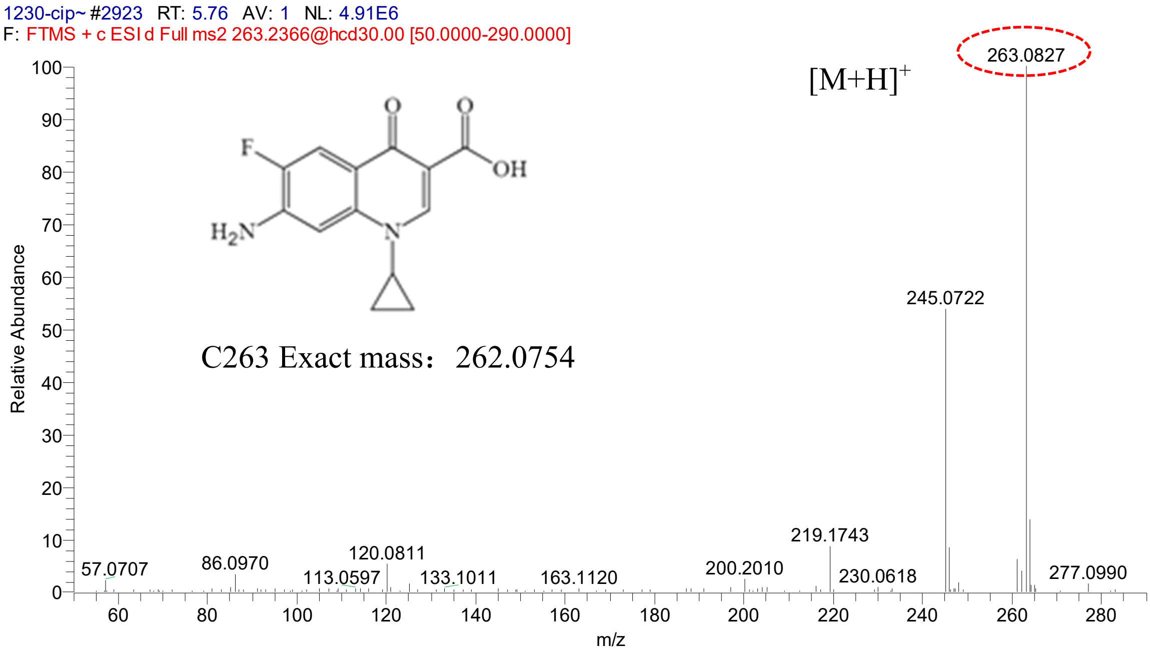

Figure S3 The MS/MS spectra of biodegradation product C263

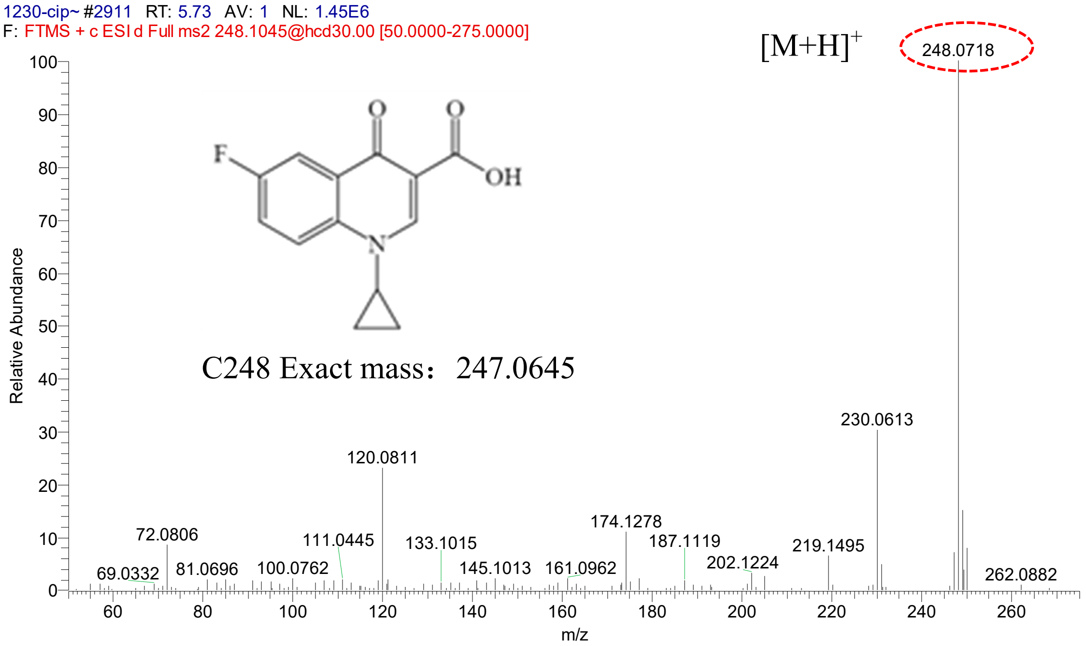

Figure S4 The MS/MS spectra of biodegradation product C248
